# Oleuropein enhances radiation sensitivity of nasopharyngeal carcinoma by downregulating PDRG1 through HIF1α-repressed microRNA-519d

**DOI:** 10.1186/s13046-016-0480-2

**Published:** 2017-01-05

**Authors:** Ting Xu, Dajiang Xiao

**Affiliations:** Department of Otorhinolarynogology, The Second People’s Hospital of Wuxi, Nanjing Medical University, 68 Zhongshan Road, Wuxi, 214002 China

**Keywords:** Nasopharyngeal carcinoma, Oleuropein, Radiation sensitivity, HIF1α, miR-519d

## Abstract

**Background:**

Oleuropein (OL) is a well-known anti-oxidative agent and is shown to reduce the hypoxia-inducible factor 1 α (HIF1α) protein expression after radiation. The current study investigated the effects of OL on radiation response in nasopharyngeal carcinoma (NPC).

**Methods:**

Colony formation assay was performed to compare the radiation response in vitro. Xenograft mouse model was used to study the OL effects on radiation in vivo*.* Chromatin immunoprecipitation and luciferase reporter assays were performed to identify the relations among HIF1α, miR-519d and PDRG1. Stable HIF1α or PDRG1 overexpression, and miR-519d downregulation were performed to test the radiation response both in vitro and in vivo.

**Results:**

OL strongly enhanced radiosensitivity of NPC cells both in vitro and in vivo. Chromatin immunoprecipitation and luciferase reporter assays suggested miR-519d was a direct target of HIF1α, and PDRG1 was a direct target of miR-519d. Overexpression of HIF1α or PDRG1, and downregulation of miR-519d abolished the radiation sensitizing effects of OL.

**Conclusion:**

Our study hereby demonstrates OL is a radiation sensitizing agent in NPC both in vivo and in vitro. OL treatment reduces the activity of HIF1α-miR-519d-PDRG1 pathway, which is essential to the radiosensitizing effects of OL.

## Background

Nasopharyngeal carcinoma (NPC) has a widely unbalanced endemic distribution, with the highest incidence reported in Southeast Asia [1]. Due to its anatomical location and relatively sensitivity to irradiation, radiotherapy is the primary treatment option for NPC patients without metastasis, although the clinical outcome of NPC has been hindered by resistance to radiotherapy. Thus, understanding the underlying mechanism of radiation resistance and developing the radiosensitive drugs are urgent needs for improving the local control and survival rate of advanced NPC patients.

Oleuropein (OL) is the main component of olive leaf extract, and is a well-established anti-oxidative agent [2]. Recently, OL has been extensively reported as a potential anti-cancer reagent by many researchers [3]. A recently study showed OL could reduce the protein expression of hypoxia-inducible factor 1 α (HIF1α) [4], which is a major regulator of the radiation response in cancer cells. Thus it is of great interest to investigate whether OL could exhibit a potential radiosensitizing effect on cancer cells.

MicroRNA (miRNA or miR) is a class of endogenous, 20–22 nucleotides in length, non-coding RNAs. MiRNAs can recognize and bind to the 3′ untranslated regions (UTRs) of their target mRNAs to suppress their translation or. There are many evidences suggesting miRNAs are involved in radiation response [5]. Kraemer et al. found that knockdown of either DICER or AGO2 sensitized endothelial cells to radiation [6]. In addition, overexpressions of miR-100 and miR-101 [7, 8] were found to sensitize tumors to radiation, whereas overexpressions of miR-17-92 and miR-155 [9, 10] protected tumor cells from radiation damage. Studies are needed to investigate if agents such as OL could exert its radiosensitizing effect through regulating miRNA expressions.

In our current study, we investigated the effects of OL on the regulation of radiation response in NPC cells both in vitro and in vivo. The potential underlying mechanism involving miRNA was studied as well.

## Methods

### Cell culture and transfection

The human NPC cell lines HNE1 and HONE1 were purchased from the Cancer Research Institute of Central South University, China. To generate HIF1α or PDRG1 overexpression cells, a lentivirus based stable overexpression system (Naldini and Trono, La Jolla, CA, USA) was used. Briefly, 293 T cells were co-transfected with either pCDH-EF1-MCS empty vector or pCDH-EF1-MCS-HIF1α/pCDH-EF1-MCS- PDRG1 with pVSV-G and pCMV-deltaR8.2 to make pseudoviral particles according to the manufacturer’s directions. HNE1 and HONE1 cells were then infected with lentivirus for 48 h, followed by one week selection in RPMI-1640 medium containing 2 μg/ml puromycin, 10% fetal bovine serum, 1% penicillin and streptomycin at 37 °C under 5% CO_2_.

### Clonogenic assays

To investigate the radiation effect in vitro, 1,000 cells/well were seeded onto 6-well plate and then radiated with different doses (0, 5 and 10 Gy) with a 210 kV X-ray source at 2 Gy/min. After incubated in RPMI-1640 medium with or without 200 μM OL treatment at 37 °C, 5% CO_2_ for 10 days, cells were fixed and stained with 0.5% crystal violet in 20% methanol. Colony numbers were counted, and plating efficiency and survival fraction were calculated afterwards. Survival fraction curves were determined with a linear-quadratic model [S = exp (-αD-βD^2^)] using GraphPad Prism 4.0 (GraphPad Prism, San Diego, CA, USA).

### Western blot

Cell lysates were prepared using ice-cold lysis buffer (Tris–HCl pH 7.8 20 mM, NaCl 137 mM, EDTA pH 8.0 2 mM, NP40 1% and protease inhibitor cocktail) and separated by SDS-PAGE gel. Blots were incubated with anti-HIF1α, anti-PDRG1 or anti-GAPDH antibody overnight following the antibody instructions. Anti-HIF1α, anti-PDRG1 antibodies were purchased from Cell Signaling Technology (Danvers, MA, USA); anti-GAPDH antibody was purchased from Santa-Cruz Biotechnology (Santa Cruz, CA, USA). Bands were visualized using Pierce ECL Substrate (Thermo Scientific, Waltham, MA, USA).

### Quantitative real-time PCR

Total RNA was obtained using TRIzol (Invitrogen, Carlsbad, CA, USA). The cDNA was synthesized using a SuperScript RT-PCR kit (Invitrogen, Carlsbad, CA, USA). The primers used in the current study included: *HIF1α* forward 5′-CTC AAA GTC GGA CAG CCT CA-3′, reverse 5′-CCC TGC AGT AGG TTT CTG CT-3′; *PDRG1* forward 5′-GAC CTG GAC ACC AAG AGG AA-3′, reverse 5′-GGT GCT CCT GAT CTT TCT GG-3′; *miR-519d* 5′-ACA CTC CAG CTG GGC AAA GTG CCT CCC T-3′, and 5′-CTC AAC TGG TGT CGT GGA-3′; *GAPDH* forward 5′- TGC ACC ACC AAC TGC TTA GC-3′, reverse 5′- GGC ATG GAC TGT GGT CAT GAG-3′. All expressions were normalized to *GAPDH* messenger level.

### Dual-luciferase reporter assay

Wild type or mutated 3′-UTR of PDRG1 mRNA were clone to the downstream of the luciferase reporter open reading frame, and the promoter region of miR-519d containing two HRE (HRE-wt) or the mutated version (HRE-mut) were cloned to the upstream promoter region of pGL3-enhancer plasmid using the pMir-Report vector kit (Applied Biosystems, Carlsbad, CA, USA). 10^5^/well cells transduced with indicated plasmid were seeded in 24 well plate and transfected with indicated luciferase constructs by Lipofectamine 2000 (Invitrogen, Carlsbad, CA, USA). Cells were allowed to grow 24 h after the transfection. The luciferase activities were determined with a dual-luciferase reporter assay kit (Promega, Madison, WI, USA) according to the instructions.

### Chromatin immunoprecipitation (ChIP) assay

Cells were cross-linked with 1% formaldehyde and subsequently immunoprecipitated with antibodies against HIF1α (Cell Signaling, Danvers, MA, USA). The PCR primers were designed to amplify the promoter regions containing HRE within miR-519d promoter region. A negative control with IgG was used. Fold enrichment was calculated as a ratio of amplification efficiency of the ChIP sample over that of IgG.

### MicroRNA microarray assay

Total RNA was extracted using Trizol (Qiagen, Valencia, CA, USA). Microarray assay was performed with the μParaflo™ MicroRNA microarray assay system (LC Sciences, Houston, TX, USA) according to the instruction. The images were collected using GenePix 4000B, Molecular Device and were analyzed using the Array-Pro image analysis software (Media Cybernetics, Rockville, MD, USA). The differences between signals with *p* < 0.05 were subjected to analysis and then were displayed in a heat-map using Java TreeView 1.0.13.

### MicroRNA assays

Stable cell lines expressing miR-519d inhibitor were made using the MISSION Lenti miR-519d Inhibitor kit (Sigma-Aldrich, St. Louis, MO, USA) according to manufacturer’s instructions. Expression efficiency of miR-519d-3p was determined using a mature miRNA assay kit (478986_mir, Applied Biosystems, Carlsbad, CA, USA).

### Xenograft model

**Fig. 1:**
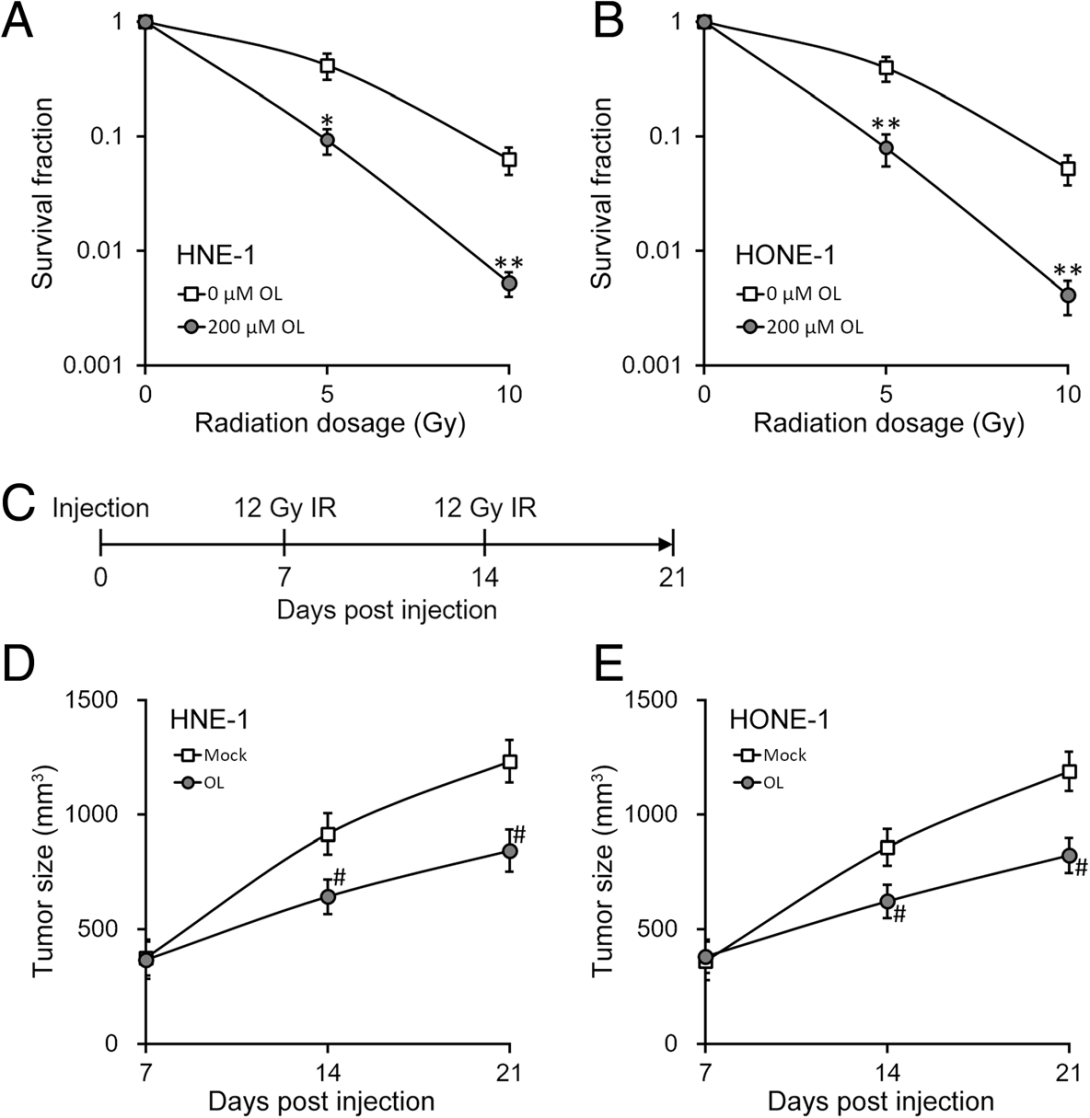
OL enhances radiosensitivity of NPC cells both in vitro and in vivo. **a** and **b** Viability of NPC cells HNE-1 (**a**) and HONE-1 (**b**) were assessed, after incubation with media containing 0 or 200 μM of OL for 24 h, respectively, and then subjected to radiation dosage as indicated. **c** Experimental time line of the xenograft mouse model, with cell injection performed on day 0 and irradiation (IR, 12 Gy) performed on both day 7 and 14. **d** and **e** Growth curve of HNE-1 (**d**) and HONE-1 (**e**) xenograft tumors in mice after mock or OL treatment, respectively (*n* = 10 each). Values were expressed as mean ± SD. * *p* < 0.05, ** *p* < 0.01, compared to 0 μM OL. # *p* < 0.05, compared to mock treatment

10^6^ /100 μl indicated tumor cells were injected to 6–8 weeks old BALB/C nude mice purchased from the Shanghai Experimental Animal Center (Shanghai, China) subcutaneously, 10 mice for each group. Tumor-injection mice were allowed to grow 7 days without any treatment to establish tumors. OL was added in drinking water at the final concentration of 1% [3] starting from day 7 to the end of the animal study (day 21). All mice received 12 Gy dosage of radiation at day 7 and 14, respectively, with an X-ray generator, as illustrated in Fig. 1c. Each mouse was covered with a lead cover allowing only tumor parts to be radiated. Tumor volumes were measured along three orthogonal axes (a, b, and c) and determined as tumor volume = abc/2. This study was carried out in strict accordance with the recommendations in the Guide for the Care and Use of Laboratory Animals of the National Institutes of Health. The protocol was approved by the Committee on the Ethics of Animal Experiments of The Second People’s Hospital of Wuxi. All surgery was performed under sodium pentobarbital anesthesia, and all efforts were made to minimize suffering.

### Statistical analysis

Results are presented as means ± SD. Differences of tumor growth curves were assessed by repeated-measure ANOVA. Differences between two groups were analyzed by student’s *t*-test. All statistics analyses were performed using GraphPad Prism 6.0 or SAS 9.4 (SAS Institute Inc., Cary, NC, USA). *p <* 0.05 was considered statistically significant. All statistical analysis was performed using SPSS 17.0 (SPSS, Inc., Chicago, IL, USA).

## Results

### OL enhances radiosensitivity of NPC cells both in vitro and in vivo

To investigate the effects of OL on radiation sensitivity, we performed colony formation assay in NPC cell lines HNE-1 and HONE-1 incubated with or without OL after irradiation. As shown in Fig. 1a and b, 24 h of 200 μM OL treatment significantly reduced the survival fractions in both cell lines, suggesting OL increased the sensitivity of these cells towards radiation. Furthermore, OL was shown to reduce the tumor volumes after radiation in a mouse xenograft model of NPC (Fig. 1c, d and e). Together, our data suggested OL strongly enhanced radiosensitivity of NPC cells both in vitro and in vivo.

### OL alters expressions of miRNAs and genes in NPC

**Fig. 2:**
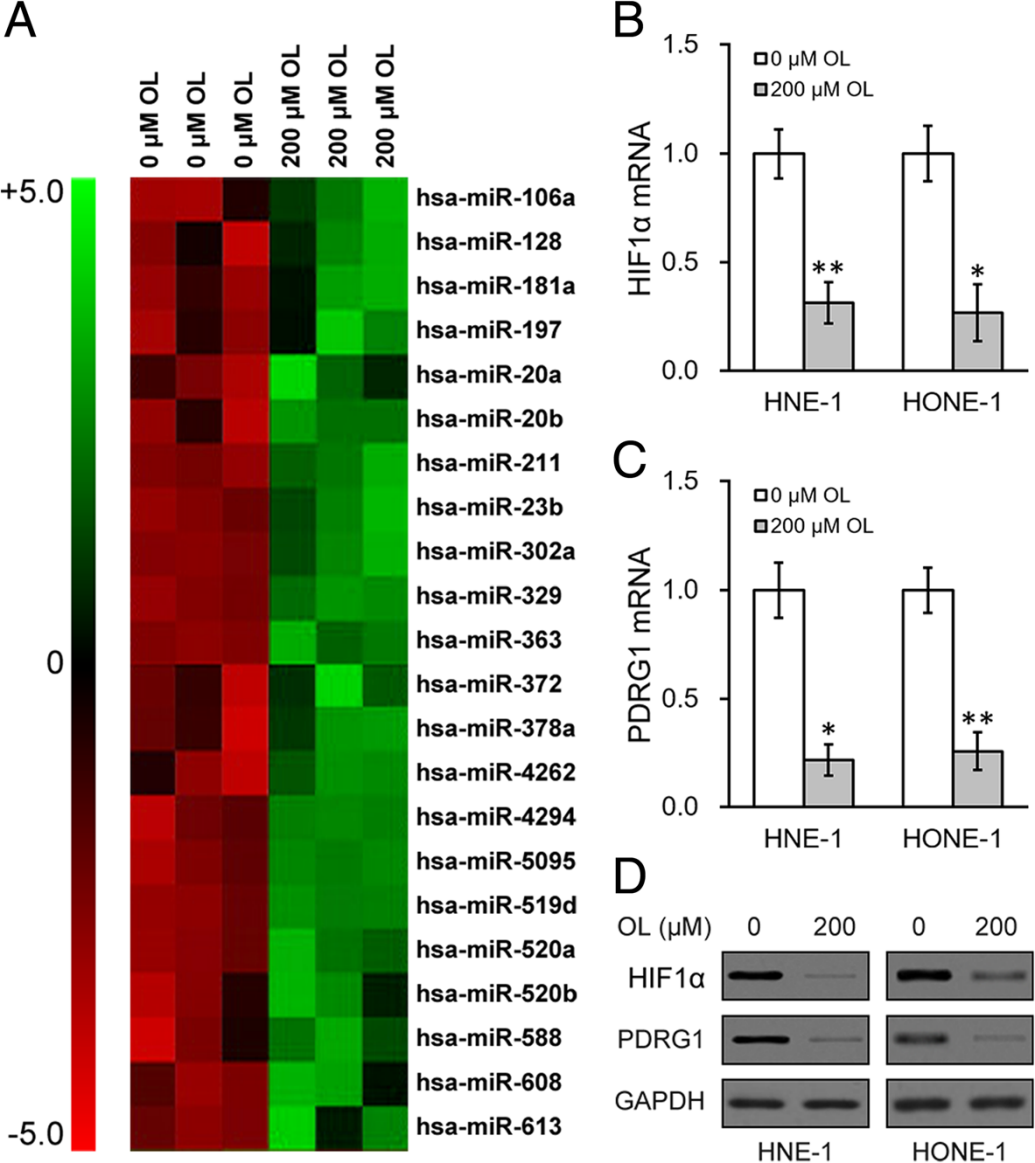
OL altered expression of genes in NPC cells. **a** NPC cells HNE-1 and HONE-1 were incubated for 24 h with media containing 0 or 200 μM of OL, respectively, and then subjected to microRNA array analysis. 22 miRNAs upregulated in both HNE-1 and HONE-1 cells were shown. **b** to **c** mRNA levels of HIF1α (**b**), PDRG1 (**c**), as well as their protein levels (**d**) were analyzed in both HNE-1 and HONE-1 cells treated as in (**a**). Values were expressed as mean ± SD. * *p* < 0.05, ** *p* < 0.01, compared to 0 μM OL

We then tried to characterize whether miRNA expression profile was altered by the OL treatment. We profiled miRNA expressions after OL treatment (Fig. 2a), and found that there were 22 miRNAs upregulated after 24 h of 200 μM OL treatment in both HNE-1 and HONE-1 NPC cell lines. Since OL is a well-known antioxidant [11], we hypothesized that OL treatment may affect the expression of HIF1α through its anti-oxidative properties. Indeed, both mRNA and protein levels of HIF1α were decreased after OL treatment in both NPC cell lines (Fig. 2b and d). Moreover, the expressions of p53 and DNA damage-regulated protein (PDRG1) were also reduced by OL treatment at both the mRNA and protein levels (Fig. 2c and d), suggesting the genes involved in DNA damage repair were repressed by OL treatment.

### MiR-519d directly targets the 3′-UTR of PDRG1 mRNA

**Fig. 3:**
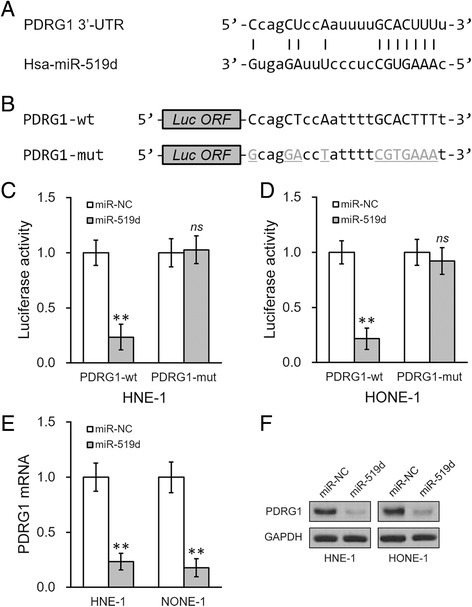
miR-519d directly targets the 3′-UTR of PDRG1 mRNA. **a** Predicted miR-519d complementing sites on the 3′-UTR of PDRG1 mRNA. **b** Wild type (PDRG1-wt) or mutated (PDRG1-mut) 3′-UTR sequences from PDRG1 mRNA were cloned at the downstream of a luciferase reporter gene open reading frame (Luc ORF). **c** and **d** The above luciferase constructs were then transfected into NPC cells HNE-1 (**c**) and HONE-1 (**d**) expressing either negative control miR (miR-NC) or miR-519d, respectively. 24 h after transfection, luciferase activities of PDRG1-wt or PDRG1-mut constructs were measured. **e** and **f** PDRG1 mRNA (**e**) and protein (**f**) levels were analyzed in cells treated as in (**c**). Values were expressed as mean ± SD. ** *p* < 0.01, compared to miR-NC

To examine whether the OL-induced upregulation in miRNA levels contributed to the decrease of PDRG1 expression, we performed an *in silicon* analysis using the miRanda algorithm [12]. Interestingly, we have identified that the 3′-UTR of PDRG1 possessed a potential binding sequence of miR-519d, which was one of the 22 miRNAs found to be upregulated after OL treatment in both cell lines (Fig. 3a). We then cloned the wild type or mutated 3′-UTR of PDRG1 mRNA into the downstream of a luciferase reporter open reading frame (Fig. 3b), which was subsequently transfected into cells stably expressing miR-NC or miR-519d, respectively. Cells were allowed to grow 24 h after the transfection followed by luciferase reporter assays. We found that overexpression of miR-519d significantly inhibited luciferase activity of wild type PDRG1 3′-UTR reporter, whereas spared the mutated versions in both cell lines (Fig. 3c and d). Furthermore, overexpression of miR-519d was sufficient to reduce the expressions of both PDRG1 mRNA (Fig. 3e) and protein (Fig. 3f) in the two NPC cell lines.

### MiR-519d upregulation by OL treatment is mediated by HIF1α

**Fig. 4:**
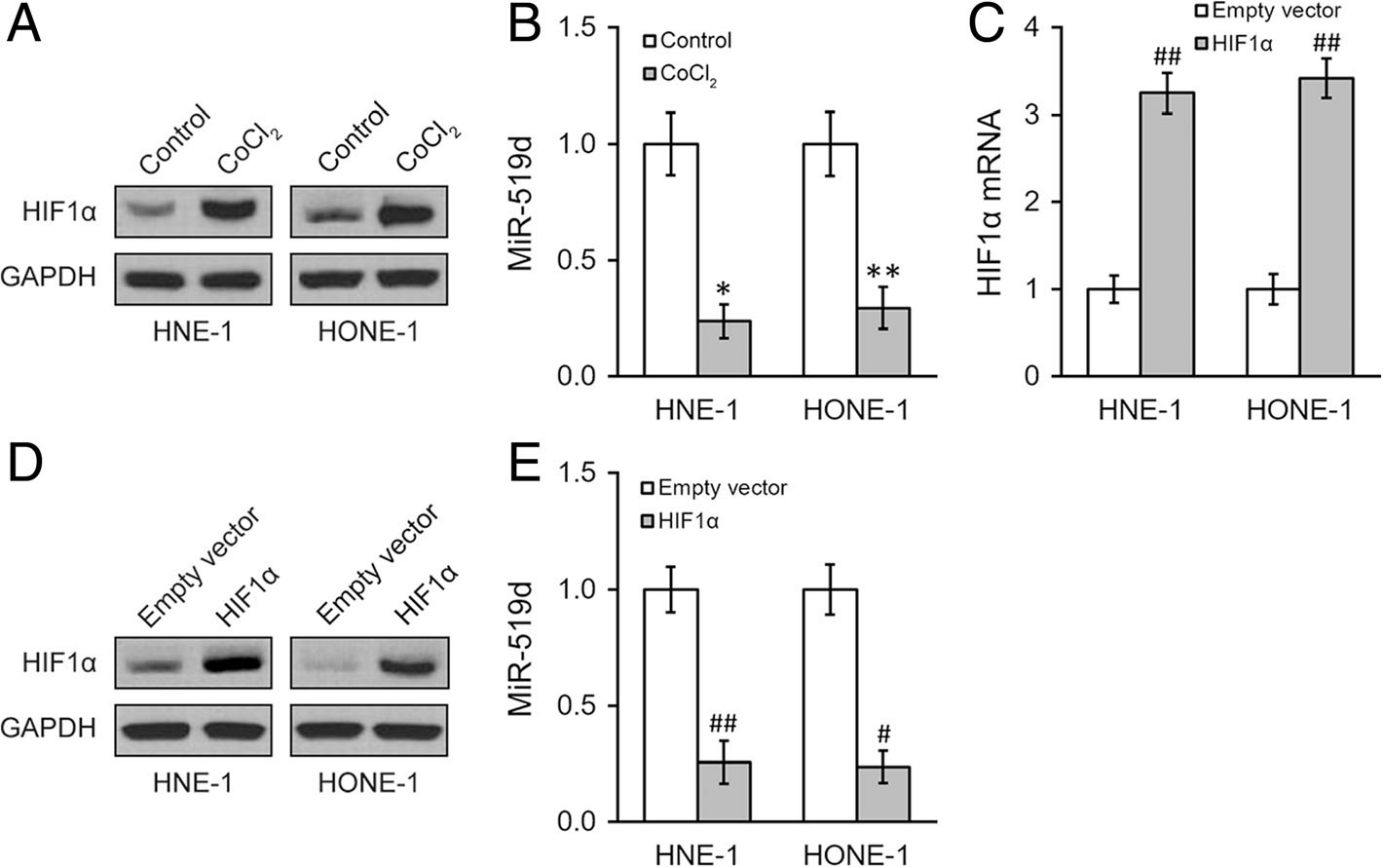
Hypoxia inhibits miR-519d expression in NPC cells. **a**, **b** NPC HNE-1 and HONE-1 were incubated for 24 h with media containing 0 or 100 μM CoCl_2_, respectively, and expressions of HIF1α protein (**a**), as well as miR-519d (**b**), were examined. **c** to **e**) HNE-1 and HONE-1 cells expressing empty vector and overexpressing HIF1α, respectively, were subjected to analyses of HIF1α mRNA (**c**) and protein (**d**), as well as miR-519d (**e**) levels. Values were expressed as mean ± SD. * *p* < 0.05, ** *p* < 0.01, compared to control. # *p* < 0.05, ## *p* < 0.01, compared to empty vector

Since OL was reported to reduce HIF1α expression [4], we tried to investigate whether the process of OL-induced miR-519d upregulation involves hypoxia and/or HIF1α itself. We treated the cells with 100 μM CoCl_2_ for 24 h to induce HIF1α expression mimicking the hypoxia condition. As shown in Fig. 4a, 24 h of 100 μM CoCl_2_ treatment was sufficient to induce upregulation of HIF1α protein. Moreover, miR-519d expressions were decreased after CoCl_2_ treatment in both cell lines (Fig. 4b). To confirm the effect of HIF1α on miR-519d expression, stable HIF1α overexpression were introduced in both cell lines using a lentivirus based stable overexpression system (Fig. 4c and d). As shown in Fig. 4e, miR-519d expressions were significantly decreased in HIF1α overexpressing cells.

**Fig. 5:**
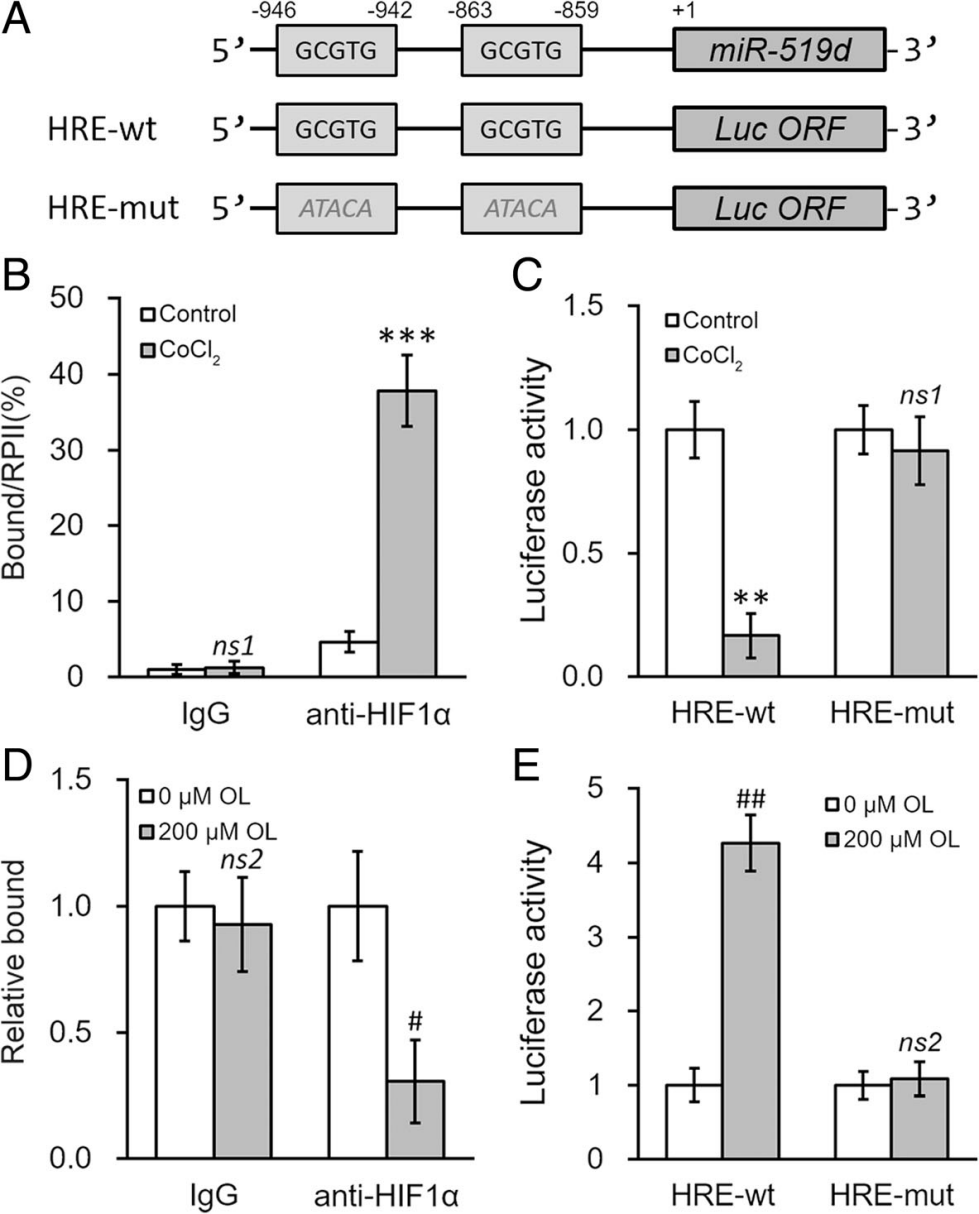
OL upregulates miR-519d expression through the hypoxia response element (HRE) in its promoter region. **a** Promoter region of miR-519d contains putative hypoxia response element (HRE). Wild type (HRE-wt, GCGTG) or mutated (HRE-mut, ATACA) HRE sites from miR-519d promoter were cloned at the upstream of a luciferase reporter gene open reading frame (Luc ORF). **b** HNE-1 cells were incubated for 24 h with media containing 0 or 100 μM CoCl_2_, respectively, followed by ChIP assay using control IgG or HIF1α antibody (anti-HIF1α). **c** Luciferase activities of HRE-wt or HRE-mut constructs were measured in HNE-1 cells treated as in (**b**). **d** HNE-1 cells were incubated for 24 h with media containing 0 or 200 μM of OL, respectively, followed by ChIP assay using control IgG or HIF1α antibody (anti-HIF1α). **e** Luciferase activities of HRE-wt or HRE-mut constructs were measured in HNE-1 cells treated as in (**d**). Values were expressed as mean ± SD. **p < 0.01, ***p < 0.001, *ns1* not significant, compared to control. #*p* < 0.05, ##*p* < 0.01, *ns2* not significant, compared to 0 μM OL

Because HIF1 complex acts as a transcriptional factor through binding to the hypoxia-response element (HRE) in various gene promoters, we checked whether the promoter region of miR-519d contained any HRE. Indeed, we have identified two HREs in the promoter region of miR-519d (Fig. 5a). Further ChIP assays using anti-HIF1α antibody showed direct binding of HIF1α to the promoter of miR-519d in NPC cells, which can be further enhanced by 24 h of 100 μM CoCl_2_ treatment (Fig. 5b). We then cloned the promoter region of miR-519d containing the two HREs (HRE-wt) or their mutated version (HRE-mut) to the upstream promoter region of pGL3-enhancer plasmid. The results of luciferase reporter assays showed decreased luciferase activity after 24 h of 100 μM CoCl_2_ treatment in cells transfected with HRE-wt, whereas HRE-mut activity was unaffected (Fig. 5c).

We next investigated whether the decrease in miR-519d expression by OL treatment was mediated by HIF1α. ChIP analysis showed that 24 h of 200 μM OL treatment reduced the binding of HIF1α to the promoter of miR-519d (Fig. 5d). The same OL treatment increased the luciferase activity in cells transfected with HRE-wt, but not HRE-mut (Fig. 5e). Taken together, our results suggested OL treatment could result in direct binding of HIF1α to the promoter region of miR-519d to induce its upregulation.

### Radiosensitizing effect of OL is mediated via the HIF1α-miR-519d-PDRG1 pathway

**Fig. 6:**
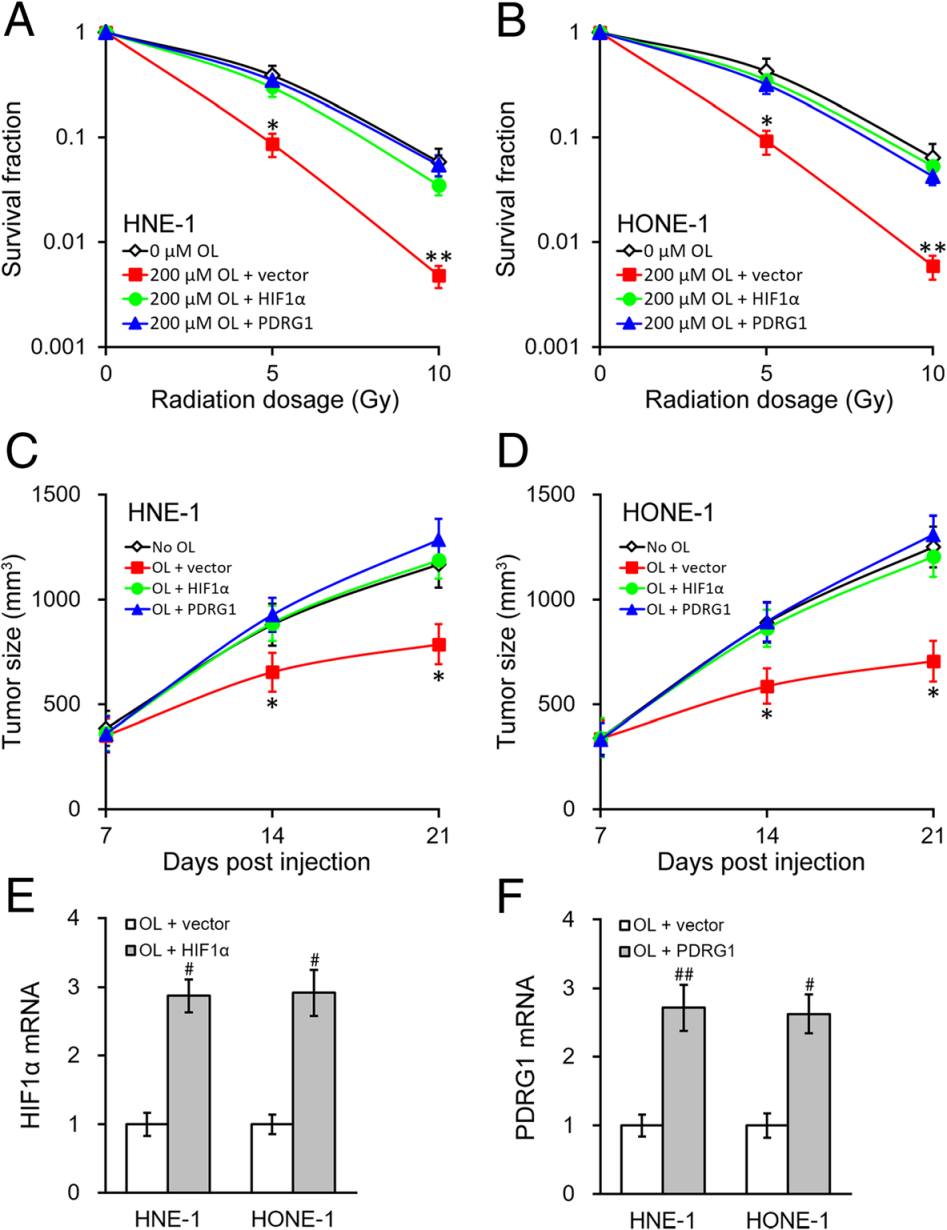
Enhanced radiosensitivity of NPC by OL can be reversed by HIF1α and PDRG1. **a** and **b** Using a lentivirus based stable overexpression system, NPC cells HNE-1 (**a**) and HONE-1 (**b**) stably expressing empty vector, and overexpressing either HIF1α or PDRG1, respectively, were incubated for 24 h with media containing 200 μM of OL, with 0 μM OL treatment as control, and then subjected to radiation dosage as indicated. **c** and **e** Growth curve of HNE-1 (**c**) and HONE-1 (**d**) xenograft tumors expressing empty vector, and overexpressing either HIF1α or PDRG1, respectively, in mice after OL treatment, with no OL administration as control (*n* = 10 each). **e** and **f** At the end of animal studies (day 21), xenograft tumors were harvested to examine the expressions of HIF1α (**e**) and PDRG1 (**f**) following respective treatments as shown. Values were expressed as mean ± SD. * *p* < 0.05, ** *p* < 0.01, compared to 0 μM/no OL, OL + HIF1α and OL + PDRG1. # *p* < 0.05, ## *p* < 0.01, compared to OL + vector

The above data of our study confirmed OL treatment could inhibit HIF1α-miR-519d-PDRG1 pathway, which significantly enhanced radiosensitivity of NPC cells. We then investigated whether the enhanced radiosensitizing effect of OL was mediated by the HIF1α-miR-519d-PDRG1 pathway. Stable HIF1α or PDRG1 overexpression was introduced in both HNE-1 and HONE-1 NPC cells using a lentivirus based stable overexpression system. As shown in Fig. 6a and b, overexpressing either HIF1α or PDRG1 abolished the radiosensitizing effect of OL treatment (200 μM OL for 24 h) in vitro, as indicated by the colony formation assays. Furthermore, the tumor sizes of the injected NPC cells with HIF1α or PDRG1 overexpression were significantly larger than those of the control cells, as shown in the xenograft mice model (Fig. 6c and d). Of note, at the end of animal studies on day 21, the xenograft tumors were harvested and analyzed for expressions of HIF1α and PDRG1 in the respective experimental groups, both of which were still greatly elevated as expected (Fig. 6e and f).

**Fig. 7:**
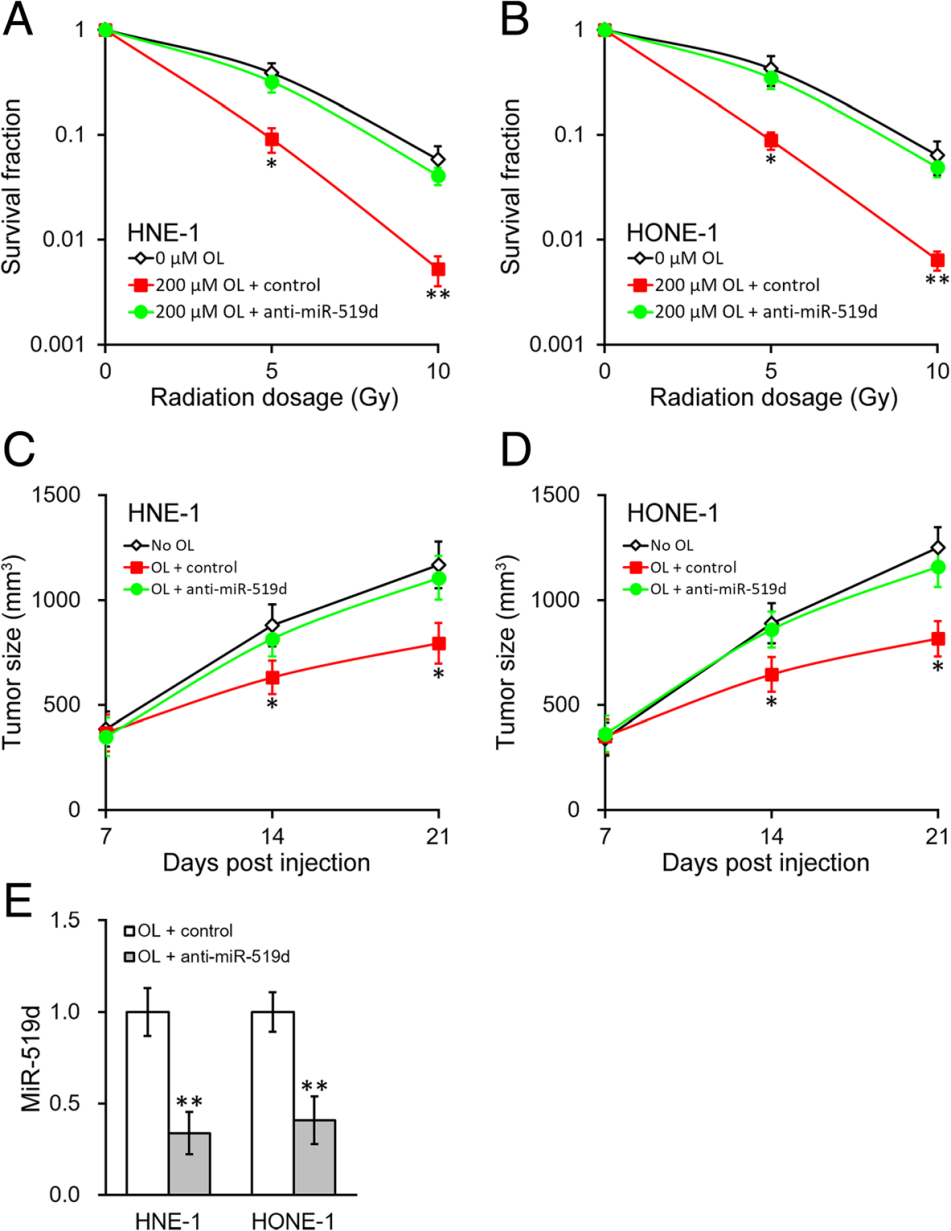
Enhanced radiosensitivity of NPC by OL requires miR-519d. **a** and **b** Using the MISSION Lenti miR-519d Inhibitor kit, NPC HNE-1 (**a**) and HONE-1 (**b**) stably expressing miR-519d inhibitor (anti-miR-519d) or control, respectively, were incubated for 24 h with media containing 200 μM of OL, with 0 μM OL treatment as control, and then subjected to radiation dosage as indicated. **c** and **e** Growth curve of HNE-1 (**c**) and HONE-1 (**d**) xenograft tumors expressing miR-519d inhibitor (anti-miR-519d) or control, respectively, in mice after OL treatment, with no OL administration as control (*n* = 10 each). **e** At the end of animal studies (day 21), xenograft tumors were harvested to examine the expressions of miR-519d following respective treatments as shown. Values were expressed as mean ± SD. * *p* < 0.05, ** *p* < 0.01, compared to both 0 μM/no OL and OL + control

At last, we examined whether miR-519d upregulation was critical to the OL radiosensitizing effect. Stable NPC cells expressing miR-519d inhibitor (anti-miR-519d) were established using the MISSION Lenti miR-519d Inhibitor kit. As shown in Fig. 7a and b, the radiosensitizing effect of OL treatment (200 μM OL for 24 h) was eliminated in the cells stably expressing miR-519d inhibitor. Similarly, the in vivo xenograft model confirmed that inhibiting miR-519d significantly increased tumor size after radiation, even with OL treatment (Fig. 7c and d). Similarly, on day 21, level of miR-519d were also examined in the harvested xenograft tumors, and was indeed efficiently repressed by the MISSION Lenti miR-519d Inhibitor kit, suggesting the long-lasting effect of the stably inhibition (Fig. 7e). Together, our results showed the HIF1α-miR-519d-PDRG1 pathway played important roles in the radiosensitizing effect induced by OL.

## Discussion

**Fig. 8:**
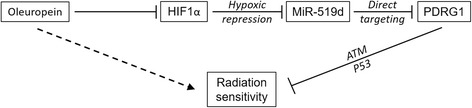
Working model of OL action in promoting radiation sensitivity. OL decreases HIF1α expression, which in turn represses miR-519d expression. miR-519d functions to directly target PDRG1 mRNA and inhibit its expression. PDRG1 is able to inhibit radiation sensitivity via the ATM-P53 axis. The overall effect of OL in NPC is then to promote radiation sensitivity

OL is the main component of olive leaf extract, and has been recognized as a healthy diet and a potential component of medicine due to its phenolic contents [2]. Previous studies have identified OL as an anti-oxidative [11, 13] and anti-inflammatory reagent [14]. Here, our current study shows that OL exhibits a radiosensitizing effect on NPC cells both in vivo and in vitro. Meanwhile, our results show OL treatment can significantly reduce the expression of HIF1α in NPC cells. Most importantly, results in our study supports the working model where OL increases radiosensitivity of NPC cells by inhibiting the activity of the HIF1α-miR-519d-PDRG1 pathway (Fig. 8).

HIF1α is the α subunit of hypoxia-inducible factor 1 (HIF1), which is an important transcription factor that mediates adaptive responses to hypoxia. The activity of HIF1 is mainly dependent on the regulation of HIF1α [15]. Under normoxic conditions, HIF1α is hydroxylated and ubiquitinated, resulting in its rapid degradation [16, 17]. Whereas under hypoxic conditions, the degradation of HIF1α is inhibited and a stable HIF1 complex is formed. HIF1α activity is also found to be significantly upregulated in various types of cancer, as a result of genetic alterations and hypoxia within the tumor tissue. The radiation and re-oxygenation of tumor tissue after radiotherapy could induce the generation of reactive oxygen species (ROS), leading to a stable HIF1 complex [18]. In addition, two downstream targets of HIF1 that can be upregulated by hypoxia, heme oxygenase-1 and stanniocalcin 2, are found to be correlated with sensitivity of NPC to radiotherapy [19, 20]. Our results are consistent with another study, showing that OL treatment caused a remarkable decrease in HIF1α protein in human colon cancer cells [4]. HIF1 pathway activation leads to induced angiogenesis and protection of tumor cells from radiation-induced apoptosis in many types of cancer. Indeed, our results show the radiosensitizing effect of OL is greatly reduced in cancer cells overexpressing HIF1α, suggesting the effect of OL is mediated via reducing HIF1α expression. Interestingly, as cellular HIF1α is mainly regulated post-translationally at the protein level, either by hypoxia or ROS [16, 17], it is intriguing that results in our current study suggest OL regulation on HIF1α occurs at the mRNA level. Further investigations are underway to reveal the biological mechanisms underlying the observed regulation of HIF1α mRNA by OL.

In the present study, we have investigated whether miRNAs are involved in OL radiosensitizing effects. MiR-519d was previously shown to sensitize ovarian cancer cells to cisplatin-induced cell death [21]. We then studied whether miR-519d was a direct target of HIF1α and involved in OL radiosensitizing effects. Our results from ChIP and luciferase assays have showed HIF1α directly binds to the promoter of miR-519d, which can be attenuated by OL treatment. Further study shows the radiosensitizing effect of OL is eliminated in the cells stably expressing miR-519d inhibitor, suggesting miR-519d is essential in the above process.

P53 and PDRG1 have been reported to be upregulated in multiple cancers including lung, breast, stomach, colon, rectum and ovary cancers [22]. PDRG1 expression was found to be correlated with higher or more advanced tumor stages, suggesting it could play a role in cancer progression [23]. A recently study also showed PDRG1 could promote radiation-resistance in lung cancer cells, which also involved the ATM-p53 signaling pathway [24]. In our current study, we have found OL treatment reduces both the expressions of PDRG1 mRNA and protein, which are necessary in the OL radiation sensitizing effects. Furthermore, through miRanda analysis, we have identified a potential binding sequence of miR-519d in the 3′-UTR of PDRG1 mRNA. Luciferase reporter assay confirms PDRG1 is a direct target of miR-519d. To our knowledge, this is the first study that reveals the relationship between miR-519d and PDRG1. Future studies are needed to investigate if other genes of the DNA damage repair mechanism are also regulated in a similar manner.

## Conclusions

In conclusion, our study hereby demonstrates OL is a radiation sensitizing agent of NPC cells both in vivo and in vitro. OL treatment reduces the activity of HIF1α-miR-519d-PDRG1 pathway (Fig. 8), which is essential to the radiosensitizing effect of OL. Further studies are warranted to optimize the regimen of OL treatment in combination with radiation therapy, and to evaluate the clinical effects of OL in NPC patients.

## Abbreviations

HIF1: Hypoxia-inducible factor 1
NPC: Nasopharyngeal carcinoma
OL: Oleuropein
UTRs: Untranslated regions
